# Supplementary material for: The Suppression of the Epithelial to Mesenchymal Transition in Prostate Cancer through the Targeting of MYO6 Using MiR-145-5p
Source: Int J Mol Sci. 2024 Apr 12;25(8):4301. doi: 10.3390/ijms25084301 (PMC11050364; doi:10.3390/ijms25084301)
Supplement: Supplementary file 1 [file ijms-25-04301-s001.zip › ijms-2947773-supplementary.pdf]

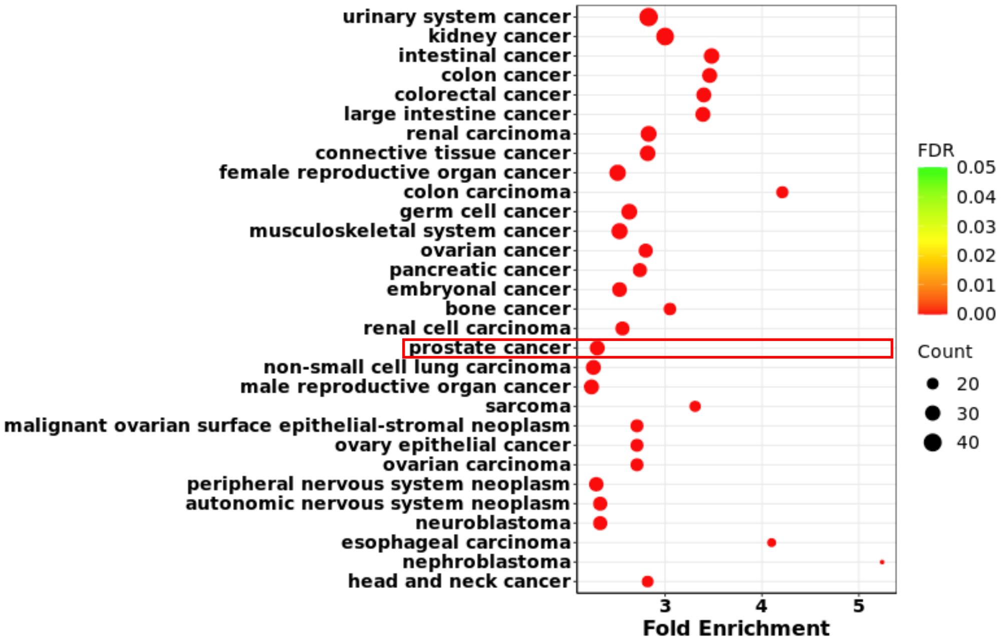

Figure S1. Bubble plot of Disease Ontology functional enrichment analysis of miR-145-5p targets. Targets of miR-145-5p significantly associated with prostate cancer.

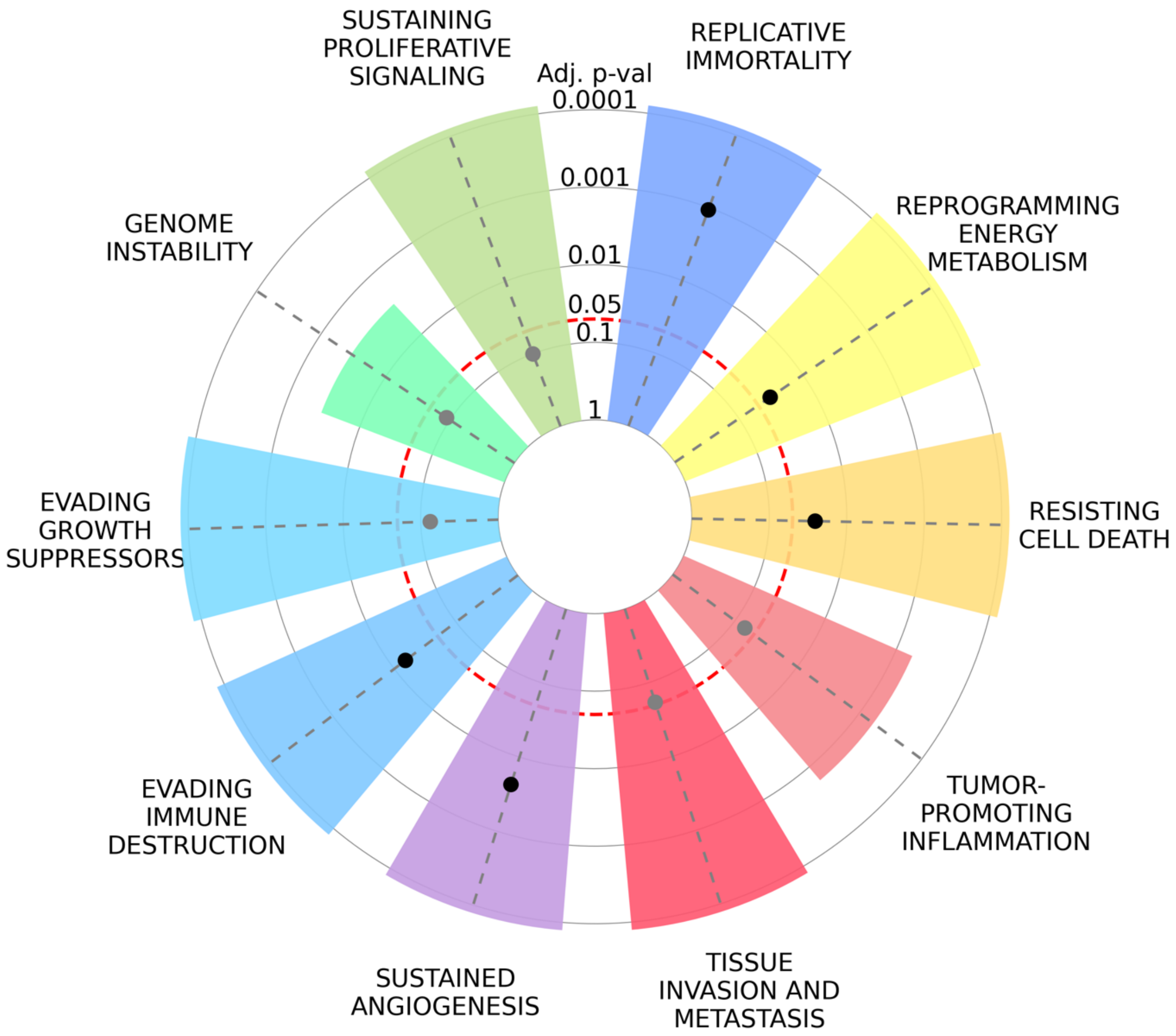

Cancer Hallmark	Overlap	*p*-value	Adjusted *p*-value	Odds Ratio	Genes
TISSUE INVASION AND METASTASIS	56/2318	1×10^{-6}	1×10^{-6}	3.96	MAP2K6;KLF4;CDH2;ITGB8;DDX17;MMP12;ROBO2;NRAS;PODXL;PXN;TGFB2;IRS1;MYC;NFATC1;SERPINE1;BRAF;PAK4;ARF6;PPP3CA;MYO6;POU5F1;CTNND1;SMAD3;MMP14;ILK;SMAD2;MMP1;COL5A1;ESR1;CD44;VEGFA;NEDD9;SOX9;CDK4;TGFBR2;HMGA2;EGFR;IGF1R;STAT1;MDM2;CDKN1A;NANOG;IRS2;SP1;ETS1;RPS6KB1;ROCK1;ADAM17;ANGPT2;CBFB;FSCN1;EPAS1;F11R;CD28;CD40;YES1

Figure S2. Enrichment analysis of miR-145-5p validated targets using integrated cancer hallmark gene set (*n* = 6763). Highlighted analysis shows significant association with tissue invasion and metastasis. Other significant hallmarks are tumour promoting inflammation, resisting cell death, reprogramming energy metabolism, replicative immortality, sustaining proliferative signalling, genome instability, evading growth suppressors, evading immune destruction and sustained angiogenesis. Generated using CancerHallmark tool (https://cancerhallmarks.com/).

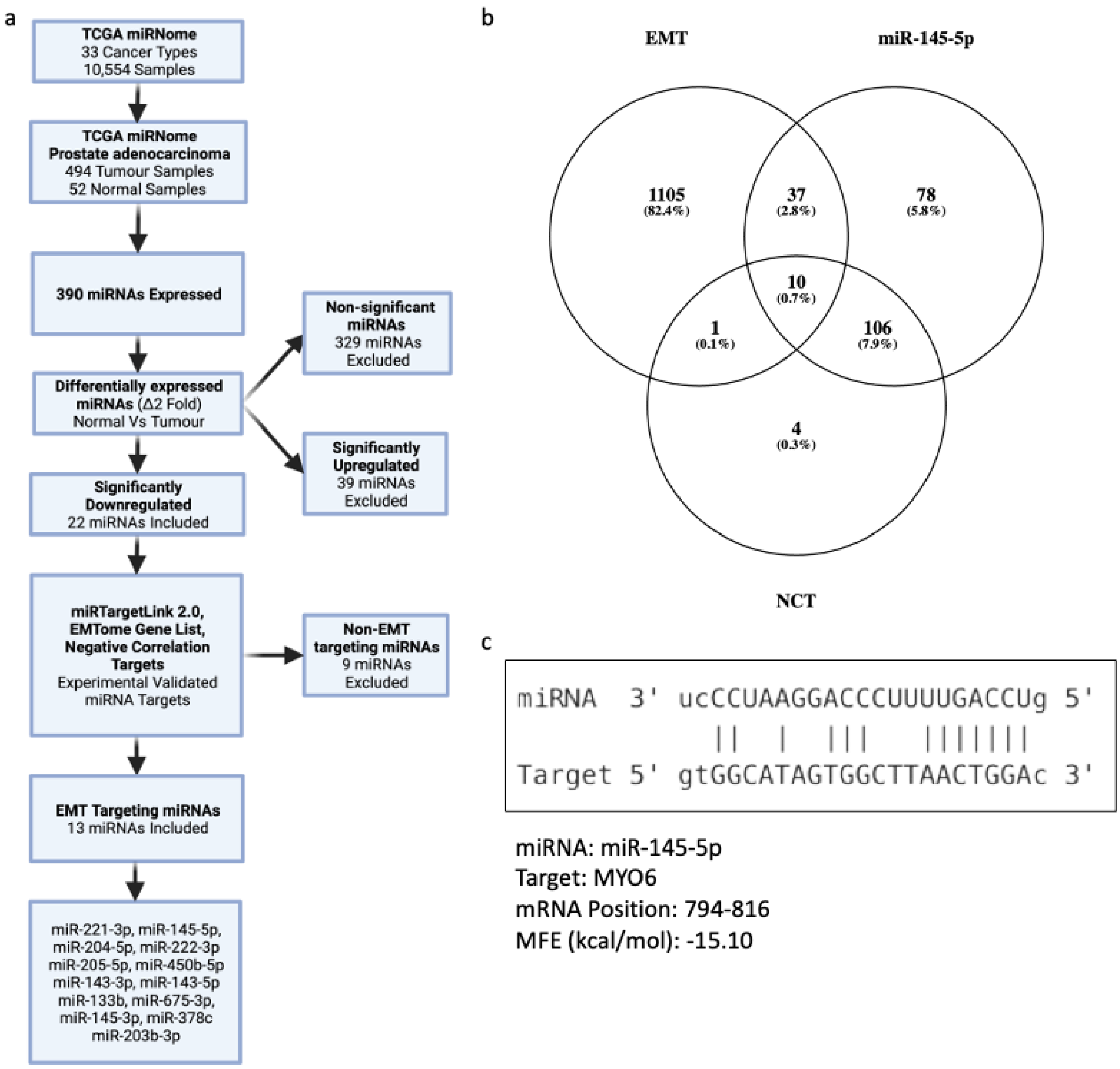

Figure S3. In silico selection of downregulated miRNAs in prostate cancer associated with EMT. (a) Selection process for identifying EMT associated miRNAs in prostate cancer. (b) Targets of the significantly downregulated miR-145-5p were predicted using miRTargetLink 2.0. Targets were cross-referenced to EMTome gene list and negative correlation targets in prostate cancer. (c) miR-TarBase computational prediction of miR-145-5p-MYO6 interaction. **EMT** = epithelial-to-mesenchymal transition; **NTC** = Negative Correlation Targets.

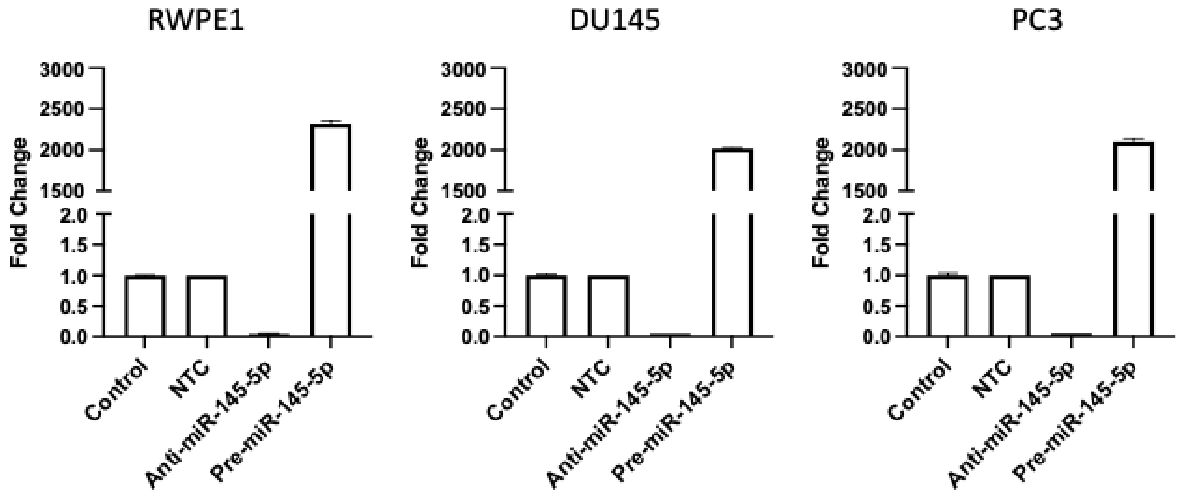

Figure S4. Confirmation of miR-145-5p overexpression and inhibition in prostate cell lines. (*n* = *3*, housekeeping: snRNA U6).

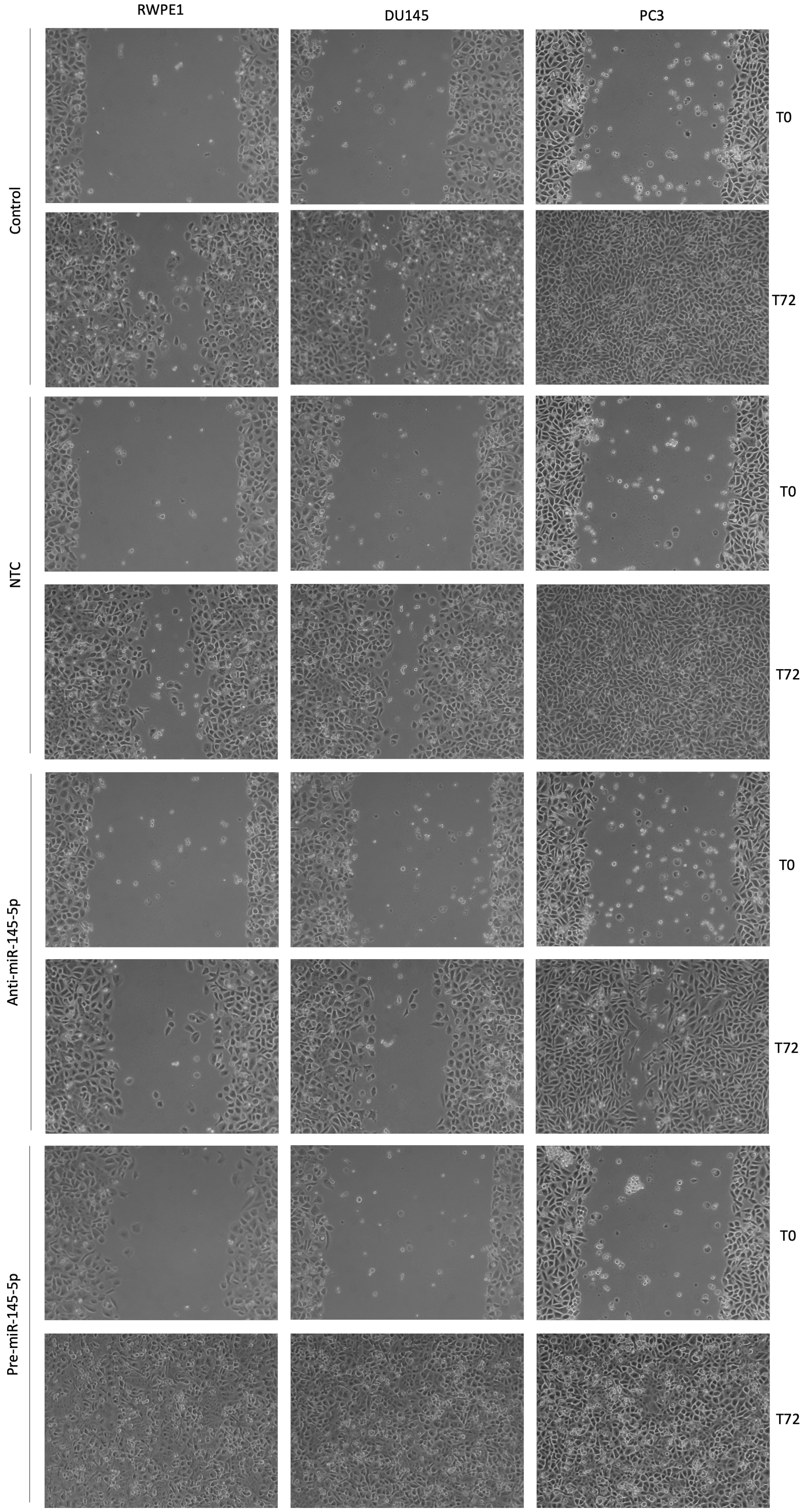

Figure S5. Representative images of wound-healing assay. miR-145-5p controls migratory capacity of RWPE1, DU145 and PC3 cell lines. Scratch assay performed at 72 hours post transfection using ImageJ software to quantify wound closure %. Pre-miR-Neg, Anti-miR-145-5p, Pre-miR-145-5p (25 nM). NTC = Non-targeting Control.

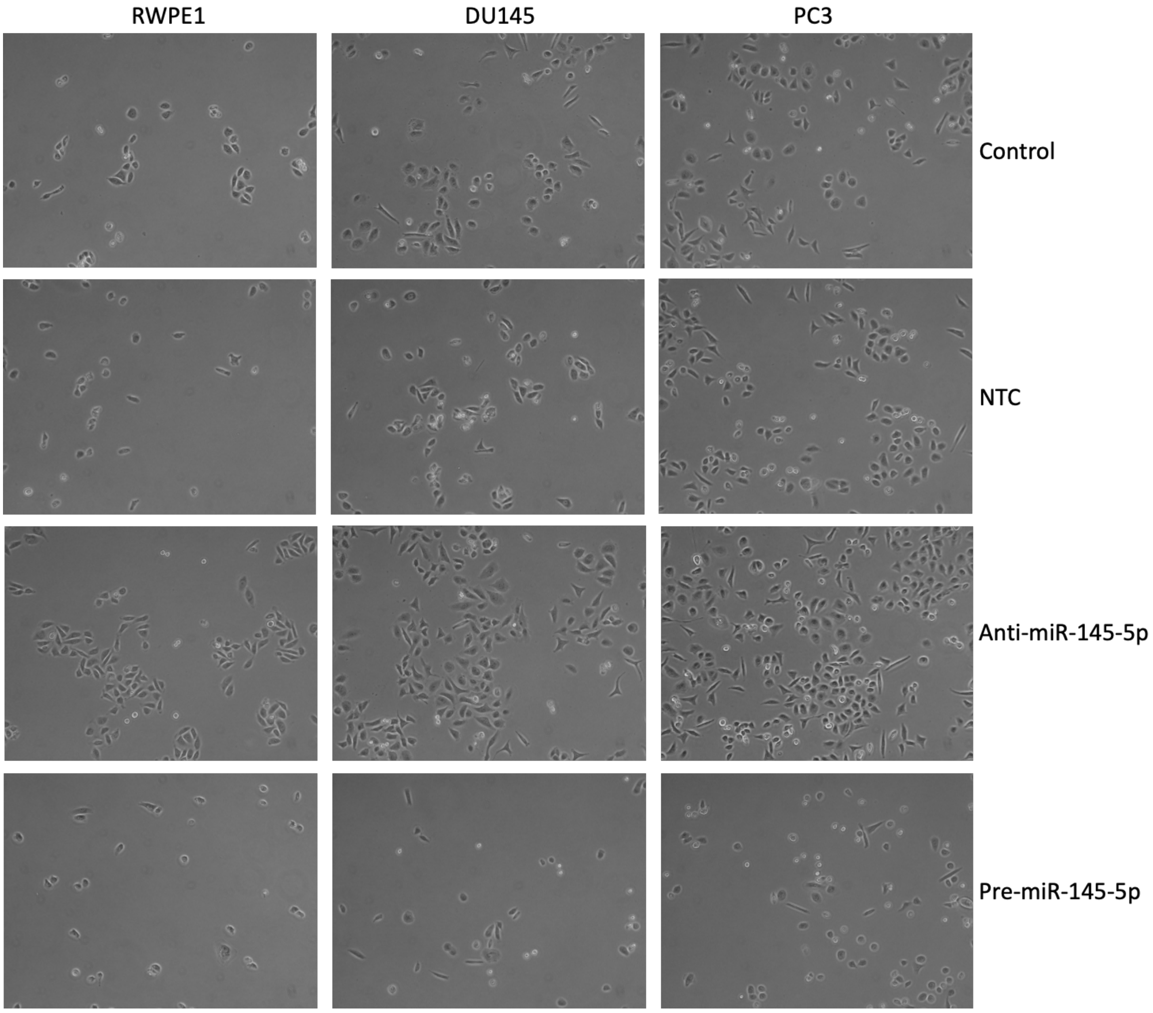

Figure S6. Representative images of clonogenicity assay. miR-145-5p alters colony forming capacity of RWPE1, DU145 and PC3 cell lines. Cell count performed using ImageJ software at 72 hours post transfection. Pre-miR-Neg, Anti-miR-145-5p, Pre-miR-145-5p (25 nM).

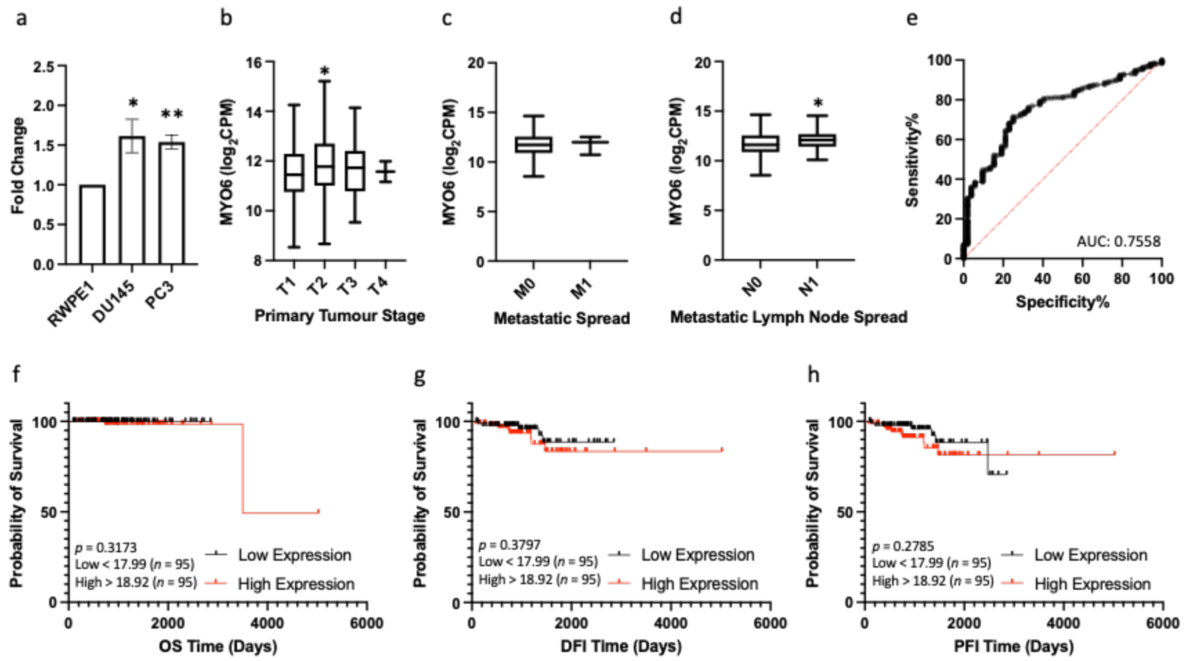

Figure S7. MYO6 upregulation is associated with prostate cancer and disease progression. (a) qRT-PCR shows MYO6 expression is significantly higher in DU145 and PC3 prostate cancer cell lines compared to normal prostate cell line, RWPE1 (n = 3, housekeeping: GAPDH). (b) pathological stage T2 (n = 292) compared to T1 (n = 197). (c) No significant difference in pathological stage M1 (n = 3) compared to M0 (n = 542) and (d) MYO6 is significant higher in pathological stage N1 (n = 80) compared to N0 (n = 385). (e) ROC curve analysis demonstrating that MYO6 shows potential for distinguishing between tumor and normal tissue in TCGA PRAD patient cohort. For KM plots, patients were divided into quartiles based on MYO6 expression for (f) Overall survival, (g) Disease-free interval and (h) Progression-free interval. (All log-rank (Mantel–Cox) test. All other p-values generated by unpaired two-tailed t-test (*$p < 0.05$, **$p < 0.01$). CPM = Copies per million; n = number.

Project	Tumor	Normal	AUC (95% CI)
TCGA-READ	161	3	1 (0.99-1)
TCGA-CESC	307	3	1 (1-1)
TCGA-UCEC	538	33	0.99 (0.98-1)
TCGA-COAD	444	8	0.98 (0.97-1)
TCGA-THYM	124	2	0.97 (0.9-1)
TCGA-LUSC	478	45	0.97 (0.96-0.99)
TCGA-KIRP	291	34	0.97 (0.95-0.99)
TCGA-BRCA	1078	104	0.97 (0.95-0.99)
TCGA-KICH	66	25	0.95 (0.91-0.99)
TCGA-SKCM	97	2	0.92 (0.79-1)
TCGA-BLCA	409	19	0.92 (0.88-0.97)
TCGA-STAD	436	41	0.89 (0.84-0.93)
TCGA-LIHC	372	50	0.85 (0.81-0.89)
TCGA-PAAD	178	4	0.8 (0.66-0.94)
TCGA-ESCA	184	13	0.77 (0.65-0.9)
TCGA-HNSC	523	44	0.73 (0.65-0.8)
TCGA-THCA	506	59	0.71 (0.65-0.77)
TCGA-LUAD	513	46	0.71 (0.65-0.77)
TCGA-KIRC	516	71	0.65 (0.59-0.71)
TCGA-PRAD	494	52	0.64 (0.56-0.71)
TCGA-PCPG	179	3	0.51 (0.29-0.72)
TCGA-CHOL	36	9	0.49 (0.29-0.7)

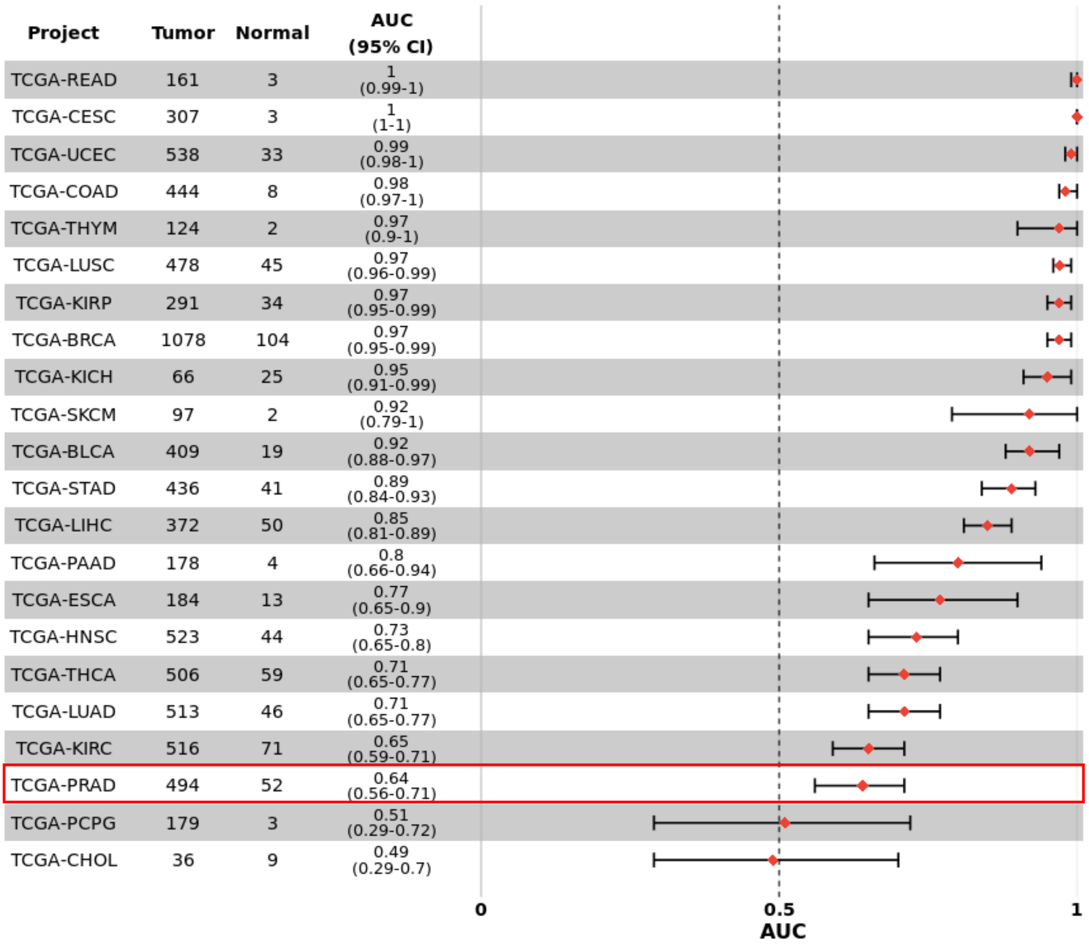

Figure S8. CancerMIRNome TCGA Pan-Cancer ranked forest plots of miR-145-5p ROC analysis shows miR-145-5p has significant diagnostic value in multiple cancer types. Result for prostate cancer is enclosed in the red box (TCGA-PRAD).

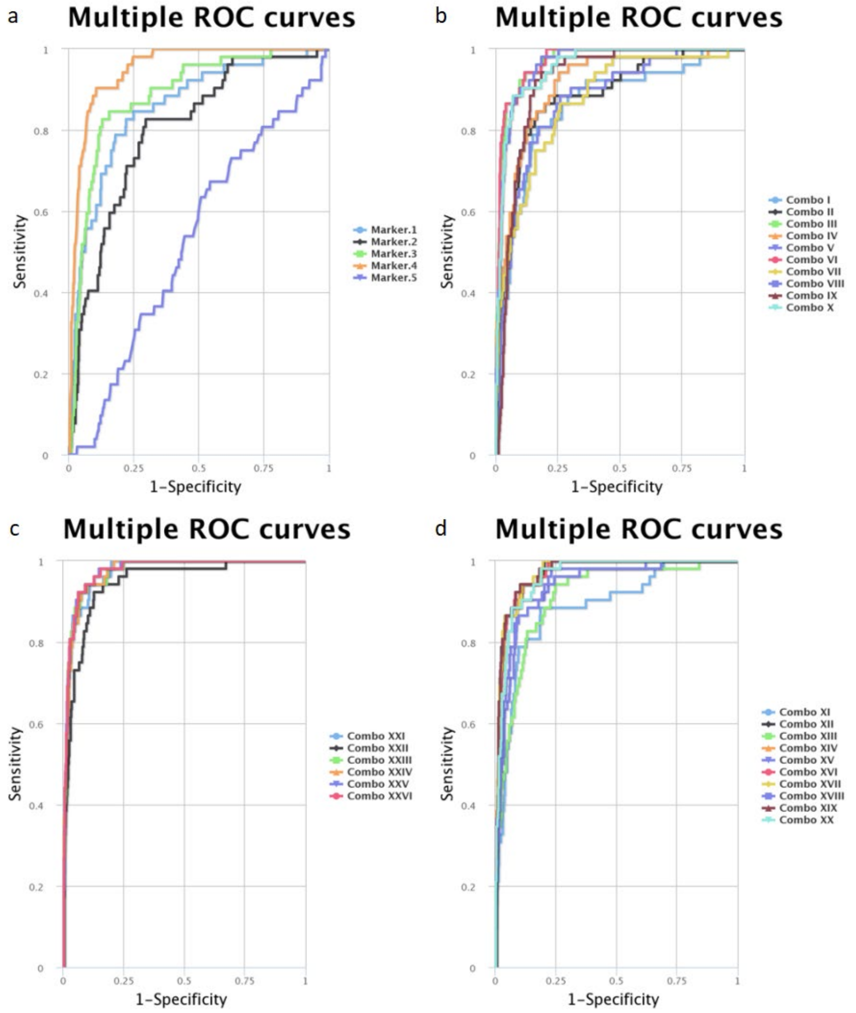

e

Key	
miR-221-3p	miR-221-3p
miR-222-3p	miR-222-3p
miR-133b	miR-133b
miR-143-3p	miR-143-3p
miR-145-5p	miR-145-5p
miR-221-3p-miR-222-3p	Combo I
miR-221-3p-miR-133b	Combo II
miR-221-3p-miR-143-3p	Combo III
miR-221-3p-miR-145-5p	Combo IV
miR-222-3p-miR-133b	Combo V
miR-222-3p-miR-143-3p	Combo VI
miR-222-3p-miR-145-5p	Combo VII
miR-133b-miR-143-3p	Combo VIII
miR-133b-miR-145-5p	Combo IX
miR-143-3p-miR-145-5p	Combo X
miR-221-3p-miR-222-3p-miR-133b	Combo XI
miR-221-3p-miR-222-3p-miR-143-3p	Combo XII
miR-221-3p-miR-222-3p-miR-145-5p	Combo XIII
miR-221-3p-miR-133b-miR-143-3p	Combo XIV
miR-221-3p-miR-133b-miR-145-5p	Combo XV
miR-221-3p-miR-143-3p-miR-145-5p	Combo XVI
miR-222-3p-miR-133b-miR-143-3p	Combo XVII
miR-222-3p-miR-133b-miR-145-5p	Combo XVIII
miR-222-3p-miR-143-3p-miR-145-5p	Combo XIX
miR-133b-miR-143-3p-miR-145-5p	Combo XX
miR-221-3p-miR-222-3p-miR-133b-miR-143-3p	Combo XXI
miR-221-3p-miR-222-3p-miR-133b-miR-145-5p	Combo XXII
miR-221-3p-miR-222-3p-miR-143-3p-miR-145-5p	Combo XXIII
miR-221-3p-miR-133b-miR-143-3p-miR-145-5p	Combo XXIV
miR-222-3p-miR-133b-miR-143-3p-miR-145-5p	Combo XXV
miR-221-3p-miR-222-3p-miR-133b-miR-143-3p-miR-145-5p	Combo XXVI

Symbol	AUC	SE	SP	Opt Cutoff
miR-221-3p	0.858	0.788	0.822	0.126
miR-222-3p	0.802	0.827	0.706	0.097
miR-133b	0.888	0.827	0.873	0.154
miR-143-3p	0.953	0.904	0.896	0.159
miR-145-5p	0.531	0.673	0.458	0.096
Combo I	0.866	0.808	0.834	0.13
Combo II	0.887	0.846	0.836	0.122
Combo III	0.972	0.923	0.906	0.1
Combo IV	0.915	0.942	0.746	0.057
Combo V	0.878	0.808	0.828	0.129
Combo VI	0.972	0.942	0.885	0.078
Combo VII	0.875	0.865	0.742	0.082
Combo VIII	0.965	0.885	0.922	0.169
Combo IX	0.922	0.923	0.843	0.124
Combo X	0.959	0.885	0.933	0.214
Combo XI	0.89	0.885	0.816	0.109
Combo XII	0.973	0.942	0.892	0.092
Combo XIII	0.915	0.827	0.871	0.133
Combo XIV	0.973	0.942	0.888	0.075
Combo XV	0.945	0.904	0.898	0.14
Combo XVI	0.973	0.923	0.92	0.134
Combo XVII	0.974	0.942	0.894	0.078
Combo XVIII	0.941	0.865	0.906	0.156
Combo XIX	0.974	0.942	0.9	0.088
Combo XX	0.964	0.885	0.935	0.23
Combo XXI	0.973	0.942	0.894	0.082
Combo XXII	0.947	0.923	0.875	0.097
Combo XXIII	0.974	0.942	0.912	0.108
Combo XXIV	0.974	0.923	0.928	0.146
Combo XXV	0.977	0.923	0.941	0.169
Combo XXVI	0.977	0.923	0.941	0.17

Figure S9. CombiROC analysis of a miRNA biomarker panel for prostate cancer detection. A combination panel of 5 miRNAs (miR-221-3p, miR-222-3p, miR-133b, miR-143-3p, and miR-145-5p) was evaluated for its ability to distinguish prostate cancer (n = 436) from normal controls (n = 48). (a) ROC curves for each individual miRNA (b-d) CombiROC curve for multiple combination panels, showing improved AUC compared to individual biomarkers. (e) Table displaying the contribution of each miRNA to the overall performance of the biomarker panel. The combination miRNA panel shows discriminatory power for detecting prostate cancer (AUC = 0.977).

Table S1. KM plotter meta-analysis results of overall survival in various cancers comparing high/low expression of MYO6 and miR-145-5p. Only cancers for which log-rank $p < 0.05$ are presented. HR estimated for high compared to low expression (Cox proportional analysis, auto-selected cut-off).

	Cancer	***n***	*HR**	*Log-rank p-value*
MYO6	Bladder Carcinoma	405	0.69	0.044
	Oesophageal Adenocarcinoma	80	2.41	0.0069
	Head-neck squamous cell carcinoma	500	0.71	0.027
	Kidney renal clear cell carcinoma	530	0.4	0.000000009
	Kidney renal papillary cell carcinoma	288	0.39	0.0062
	Liver hepatocellular carcinoma	371	1.68	0.0034
	Lung adenocarcinoma	513	0.53	0.00028
	Ovarian cancer	374	0.74	0.027
	Pancreatic ductal adenocarcinoma	177	1.45	0.078
	Rectum adenocarcinoma	165	0.39	0.013
	Thymoma	119	6.44	0.0078
miR-145-5p	Bladder Carcinoma	409	1.66	0.00085
	Cervical squamous cell carcinoma	307	0.47	0.0059
	Oesophageal Squamous Cell Carcinoma	89	3.32	0.008
	Kidney renal clear cell carcinoma	516	0.65	0.017
	Kidney renal papillary cell carcinoma	291	3.23	0.00004
	Liver hepatocellular carcinoma	372	0.64	0.019
	Lung adenocarcinoma	513	0.71	0.02
	Stomach adenocarcinoma	436	1.79	0.0014
	Thymoma	124	0.12	0.0016

* Red text indicates HR > 1; high expression of miR-145-5p or MYO6 predicted poor prognosis; blue text indicates HR < 1; low expression of miR-145-5p or MYO6 predicted favourable prognosis. *n* = number; HR = Hazard ratio; KM = Kaplan-Meier
